# Accidents of Electrical and Mechanical Works for Public Sector Projects in Hong Kong

**DOI:** 10.3390/ijerph15030485

**Published:** 2018-03-10

**Authors:** Francis K. W. Wong, Albert P. C. Chan, Andy K. D. Wong, Carol K. H. Hon, Tracy N. Y. Choi

**Affiliations:** 1 Department of Building and Real Estate, The Hong Kong Polytechnic University, Hung Hom, Kowloon, Hong Kong, China; francis.wong@polyu.edu.hk (F.K.W.W.); albert.chan@polyu.edu.hk (A.P.C.C.); andy.wong@polyu.edu.hk (A.K.D.W.); 2 School of Civil Engineering and Built Environment, Queensland University of Technology, 2 George St., Brisbane, QLD 4001, Australia; carol.hon@qut.edu.au

**Keywords:** electrical and mechanical, safety, public sector projects, cluster analysis

## Abstract

A study on electrical and mechanical (E&M) works-related accidents for public sector projects provided the opportunity to gain a better understanding of the causes of accidents by analyzing the circumstances of all E&M works accidents. The research aims to examine accidents of E&M works which happened in public sector projects. A total of 421 E&M works-related accidents in the “Public Works Programme Construction Site Safety and Environmental Statistics” (PCSES) system were extracted for analysis. Two-step cluster analysis was conducted to classify the E&M accidents into different groups. The results identified three E&M accidents groups: (1) electricians with over 15 years of experience were prone to ‘fall of person from height’; (2) electricians with zero to five years of experience were prone to ‘slip, trip or fall on same level’; (3) air-conditioning workers with zero to five years of experience were prone to multiple types of accidents. Practical measures were recommended for each specific cluster group to avoid recurrence of similar accidents. The accident analysis would be vital for industry practitioners to enhance the safety performance of public sector projects. This study contributes to filling the knowledge gap of how and why E&M accidents occur and promulgating preventive measures for E&M accidents which have been under researched.

## 1. Introduction

Accident analysis plays an important role in safety management. It provides invaluable lessons for causes of accidents and helps to formulate effective preventive strategies. For instance, Haslam et al. [1] studied the contributing factors in construction accidents. Hon and Chan [2] explored the fall fatalities of repair, maintenance, minor alteration, and addition (RMAA) works. Wu et al. [3] investigated struck-by falling object accident cases and developed an integrated information management model for preventing struck-by falling object accidents in construction sites proactively. Tam and Fung [4] explored the key factors of tower crane safety and the related statutory and non-statutory guidelines for using tower cranes. Lingard et al. [5] undertook a detailed investigation into the causes of fatalities involving plants such as excavators and trucks. However, the accidents on electrical and mechanical (E&M) works, as a standalone category of activity, has not been fully investigated.

E&M work is an essential work category in both new construction works and RMAA works. Understanding the underlying relationship between E&M works-related injuries and factors leading to the injuries are important to enhance E&M work safety. E&M works involve a wide range of building services trades such as air-conditioning, fire services, plumbing and drainage, electrical wiring and lift installation and maintenance work. E&M safety is a vital issue in promoting construction safety. Literature has demonstrated that electrical work-related accidents are serious in construction [6,7]. Electrical hazards are regarded as one of the major fatal hazards to construction health and safety in the United States [7]. Electrical fatalities in Taiwan were the second leading causes of occupational fatality, following falling fatalities [8].

According to Census and Statistics Department [9], the number of persons directly engaged in “building services installation and maintenance activities” in 2014 was 66,592, representing around 35% of the number of persons directly engaged in “all construction activities” (*n* = 189,454). Under the “Construction Workers Registration Ordinance” which was established in 2005, a construction worker must be registered before carrying out work on construction sites. By the end of December 2015, 47,104 E&M trade workers had been registered, contributing to more than 35% of all skilled workers. In terms of the number of “building services installation and maintenance activities” establishments, it was 7669 and accounts for 31% of the number of establishments of “all construction activities” (*n* = 24,864). With this significant number of registered E&M workers and companies involved, the E&M sector represents a major player in the construction industry. However, despite its importance, accident statistics exclusively for E&M activities are not available in the public domain. Safety research on E&M works regardless the type of works (i.e., new construction work, repair, maintenance, alteration, addition (RMAA) works, public sector and private sector projects), is very limited and the safety of E&M works seems to have been overlooked.

Architectural Services Department (ArchSD) and the Electrical and Mechanical Services Department (EMSD) are the two core government works departments in Hong Kong managed under the Development Bureau (DEVB). The main services of ArchSD are to develop and construct government-owned and government-funded buildings and related facilities [10]. It also provides services for the repair, maintenance and refurbishment of buildings and facilities. The EMSD provides trading services to more than a hundred government departments and organizations, public institutions and the community with electrical and mechanical installation and maintenance services [11]. The services of ArchSD and EMSD contribute to a significant portion of government E&M-related works. The “Public Works Programme Construction Site Safety and Environmental Statistics” (PCSES) system, an accident database, is established and maintained by the Development Bureau for recording accident statistics of public works contracts. All reportable accidents resulting in death and any severity level of injury with incapacity for more than three days are to be reported and inputted in the PCSES system [12].

The aim of this study is to explore the causes of electrical and mechanical accidents in the data set of the PCSES system from the ArchSD and the EMSD over 15 years from 2001 to 2015. The significance of this study lies in providing a systematic E&M accident analysis in the public sector, for the first time. This study contributes to filling the research gap of analyzing accidents on E&M-related works. Findings are useful to E&M practitioners and safety professionals to better understand how and why accidents happened in E&M work and to therefore better promulgate appropriate preventive measures. The paper starts with a concise literature review of E&M-related research and the characteristics of E&M works. The methodology of the research is then outlined, which is followed by the research results and discussions. The research outcomes of this study could establish recommendations for preventing reoccurrence of E&M accidents.

## 2. Previous Research

A variety of construction safety management research focusing on areas such as safety culture, design for safety, safety climate and worker behavior has emerged. A systematic review on safety management studies by Zhou et al. [13] identified that over 90% of the safety management research studies in the construction industry have concentrated on industry, company and project levels. Wilson and Koehn [14] investigated the safety management philosophy applied to small and medium-sized projects in the United States. Nguyen et al. [15] studied success factors and success criteria for large construction projects in Vietnam. Some studies focused on the relationship between construction safety and size or type of company [16,17,18,19]. Studies at sub-project level such as excavation, electrical and mechanical, piping and steel work has been scant. As individual tasks are the basic components of any construction project, ensuring the safety of the different individual tasks is an essential prerequisite for safety on site. Electrocution is one of the most common types of fatal accident in most construction industries. Therefore, some studies on electrical injuries and electrical safety for workers have been conducted. Janicak [20] examined the occupational fatalities due to electrocutions in the U.S. construction industry between 2003 and 2006. The study shows that the proportion of fatalities due to contact with electric current is significantly higher for younger workers in 16 to 19 years old age group. Chi et al. [8] conducted an in-depth accident analysis of 255 electrical fatalities in Taiwan and categorized the incidents in terms of the cause, performing task, individual factor, company size and source of injury for developing effective electrical protection strategies. Inexperienced workers and those working for smaller companies were found to be at the greatest risk of electrocutions. Zhao et al. [7] explored the 486 control measures of electrical hazards to construction workers through quantitative and qualitative analysis of 134 electrocution case reports from 1989 to 2012. The research findings revealed that behavioral controls of workers remain prevalent in control of electrical hazards. While there have been individual studies on electrocution fatalities [8,13,20,21], safety research on air-conditioning, lift installation, fire services, and plumbing works has been lacking. Due to the complexities and characteristics of E&M works, safety risks vary a lot accordingly. Because the safety risks of E&M works are often different from and higher than building works, there should be an independent study to explore the “how and why” of E&M works-related accidents.

## 3. Characteristics and Risk of Electrical and Mechanical Works

Electrical and mechanical (E&M) installations are key activities in all construction works and indispensable to any building types. E&M works includes installation and maintenance of air-conditioning system, electrical wiring, lift and escalators, fire services system and plumbing and drainage system. E&M works involves lots of high-risk activities, for examples, works often involve electricity, confined working space, lifting, machinery (for lift and escalator), welding, using handheld tools, etc. Some hazards are quite particular to E&M work processes such as lifting of chillers and generators, electrical hazard in switch gear work, confined space hazards at water tank, etc. Electrocution is also a major type of E&M-related accident. A tight construction schedule for E&M works is one of the most significant characteristic in new construction works. As the installation of E&M works needs to follow the general builder’s works, the delay of previous construction works will carry on to the E&M works. Even though E&M works are postponed due to delay of completion of the general builder’s works, E&M contractors still need to strictly follow the master programme because extra time is not typically allowed. The condition of a new construction site is generally untidy and with unforeseeable working situations. Several building service trades are often concurrently working at the same location [22]. The coordination of multiple trades is important for the complex working environment for E&M works, particularly electrical installations. Electrocution is a major type of E&M-related accident. For electrical wiring works, the E&M workers are vulnerable to electric shock hazard whilst working on the conductive parts of the electrical cable which has not been properly isolated from the power source. The main switch should be properly isolated, locked out, and verified dead by voltage indicator or suitable testing equipment to prevent accidental energization or interference of electrical circuit by other workers. E&M accidents of lift installation and maintenance works are usually caused by a variety of factors such as lack of safety access to lift pit, inappropriate location of cat ladder in lift pit, inadequate lighting and poor ventilation. E&M workers may be injured by moving machinery when there is no separation in the lift well. Moreover, cat ladder is commonly used to access the lift machine room. It would be dangerous for workers to climb up a cat ladder with heavy tools, equipment and materials.

E&M RMAA works generally last for a short construction period with less safety resources, equipment and inadequate safety supervision (i.e., without safety personnel). As repair and maintenance of air conditioning systems, electrical wiring, water pipes and fire services always involve working at height, fall injuries frequently occur when using ladders. Wong et al. [23] pointed out that four factors, namely, inappropriate equipment, lack of design for safety, lack of resources and insufficient housekeeping, are the main factors contributing to fall injuries. For E&M works, the key hazards are identified in activities that involve working at height, with electricity, in confined working spaces, lifting, machinery (for lift and escalator), welding, and using handheld tools, etc. Some hazards in E&M works are quite unique, such as the E&M work processes in lifting of chillers and generators, electrical hazards at switch gear works, and confined space hazards around water tanks, etc.

To formulate effective strategies to prevent E&M-related accidents, a full investigation into the contribution factors of E&M accidents and corresponding improvement measures is urgently needed. This requirement has not received the committed level of attention it deserves. This study is a much-needed contribution towards filling this research gap.

## 4. Methodology

### 4.1. Data Source

Accident data should be properly recorded, maintained and analyzed to indicate where, when and how the accidents arise. Accident prevention measures can then be established to focus on the problem area. According to the chapter 9 of the Construction Site Safety Manual [12], the safety officer or site agent of the principal contractor should complete the injury report form (version 2001) and submit it to the Departmental Safety and Environmental Advisory Unit within seven days on occurrence of accident for entry into the PCSES system. The unsafe action, unsafe condition and personal factor which causes the accident are then assessed by qualified safety personnel.

With the consent of the Development Bureau, a total of 421 sets of accident cases related to E&M works for the period between 2001 and 2015 were provided by the Electrical and Mechanical Services Department (EMSD) and the Architectural Services Department (ArchSD) of the Hong Kong Government. It was decided to exclude the accident data before 2001, since the injury report form was revised significantly in 2001. Based on the injury report form, an EXCEL file with 18 variables describing the characteristics of the accident was developed for data input. The variables used in the analysis are summarized in Table 1.

### 4.2. Cluster Analysis

To examine the typology of E&M works accident cases, cluster analysis was adopted in this study. Cluster analysis is regarded as a multivariate statistical technique for grouping cases of data based on their homogeneity [24,25]. It was adopted by [2] to investigate fall fatalities of RMAA works in Hong Kong. Three cluster groups of fall fatalities were identified. In addition to construction-related research, this technique was used for accident analysis in various research areas such as transportation safety and pedestrian safety [26,27,28]. In this study, the accident cases can be grouped on the basis of the injured workers’ personal experience, working conditions and working behavior patterns. This segmentation of case helps in developing safety strategy for different segments of E&M workers. It is useful to identify distinct groups of E&M workers so that safety preventive measures can be appropriately targeted. A total of 421 accident cases inputted in the EXCEL template and analyzed by using SPSS 21 (SPSS Inc., Chicago, IL, USA). There are three methods for the cluster analysis—K-means cluster, hierarchical cluster and two-step cluster; all can be found in SPSS. Two-step cluster analysis, which is a log-likelihood distance criterion measure used for identifying the grouping of cases within a large data set, was adopted in this research. As this clustering procedure is effective for handling both mixtures of continuous (i.e., age, year of experience) and categorical variables (i.e., sex, trade of E&M works) [29], two-step cluster was adopted in the current research. It also provides automatic identification of the optimal number of clusters present in the data [24,29,30]. Two-step cluster analysis is comprised of pre-cluster formation and hierarchical clustering of pre-clusters. The aim of pre-clustering is to reduce the size of the matrix that contains distances between all possible pairs of cases. Based on the distance between the cases, it decides if the current case should be merged with a previous formed pre-cluster or starts a new pre-cluster. The standard hierarchical clustering algorithm is applied on the pre-clusters to obtain a range of cluster solutions with different numbers of clusters in the second step [30]. To determine which number of clusters is the best, Norusis [31] suggested that Schwarz’s Bayesian criterion (BIC) can be used as the clustering criterion. In the final stage, Chi-square test was used for testing whether the variables differ significantly across all the identified cluster of E&M works-related accidents. All calculated *p*-values are two-tailed, and the null hypothesis of no significant difference was rejected at the significance level at <0.05.

## 5. Research Results and Discussion

### 5.1. Descriptive Statistics

Background information of the 421 E&M works-related accident cases are shown in Table 2. 170 (40.4%) E&M works-related accident cases were collected from the Electrical and Mechanical Services Department (EMSD), and 251 (59.6%) from the Architectural Services Department (ArchSD) of the Hong Kong Government (Table 2). As most of the construction works are physically demanding and performed in outdoor environments, the construction industry is very much male-dominated [32]. It is not surprising that a vast majority of the injured workers were male and only three (0.7%) injured workers were female. The vast majority (97%) of injured workers were non-imported labourers while only 1.4% of them were imported labourers. Over 73% (*n* = 308) of the E&M work-related accidents occurred in RMAA works which far outweighed that of new construction works. This may be explained by the fact that the working conditions of new construction works is comparatively better than RMAA works. The construction processes of new works are relatively well-planned whereas those of RMAA works are rather unforeseeable [33]. RMAA works last for a shorter construction period with less safety resources and safety supervision (e.g., employment of safety officer or supervisor).

The number of E&M accidents the occurred in summer (*n* = 134) was significantly higher than other seasons, especially in autumn and winter, accounting for about 31% of the 421 accident cases. The high temperature and humidity environment with low wind speed is insufferable and unfavorable to safety and health of construction workers [34]. It is believed that prolonged work in a hot environment may result in fatigue, heat-related illness and a higher chance of injury [35,36]. It seems that hot and humid weather in summer is a key contributing factor for E&M works-related accidents. Over 36% E&M accidents occurred at the beginning of the week (i.e., Monday and Tuesday) while relatively less accidents happened in the end of the week and weekends. This result was consistent with the findings of Camino López et al. [37] that construction accidents on Monday were more frequent than on other days within a week.

**Figure 1:**
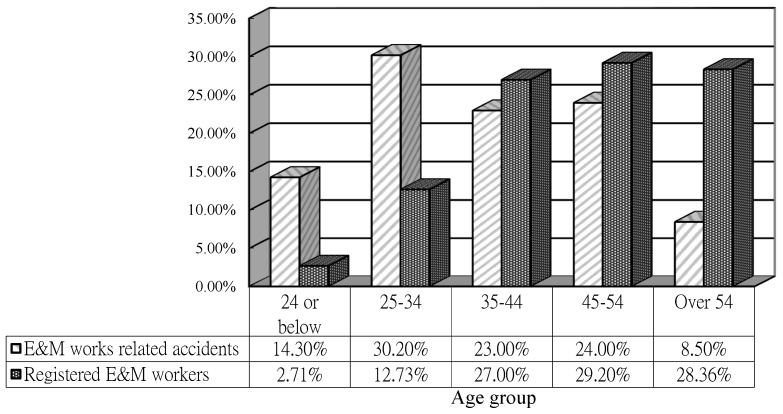
Age distribution of Registered E&M Workers (as at 30 September 2015) and E&M accident data analysed by age [38].

With the “Construction Workers Registration Ordinance” having taken effect from December 2005, all workers carrying out construction works on construction sites must be registered under the Construction Workers Registration Authority [38]. Workers in E&M installations need to be registered as “Registered Skilled Worker” for their own trades. Referring to Figure 1, workers aged 34 or below accounted for 44.5% of the E&M work-related accidents collected in this study, whereas registered E&M workers aged 34 or below only accounted for 15.44% of the registered E&M workers in Hong Kong as on 30 September 2015. It is probable that young E&M workers were more prone to accidents due to inexperience. The findings are in line with Chi et al. [39] and Salminen [40] that young workers had a higher non-fatal injury rate than older workers. The research of Choudhry and Fang [18] further pointed out that the most effective safety training for new construction workers is learned by doing or by gaining experience. New workers become more aware of construction safety when they accumulate more working experience.

The results also indicated that the majority of injured workers had less than five years of working experience in construction (*n* = 136, 32.3%), and work at that construction site with not more than three months (*n* = 220, 52.3%) (Table 3). Among these 220 accident cases, over half of them (*n* = 128) involved workers engaged in that construction for not more than one month. As construction is always risky due to its complexity and continuously changing working environment as well as the associated hazardous characteristics of E&M works, new workers who are less familiar with the site and working environment are more prone to accidents.

### 5.2. Cluster Analysis

**Figure 2:**
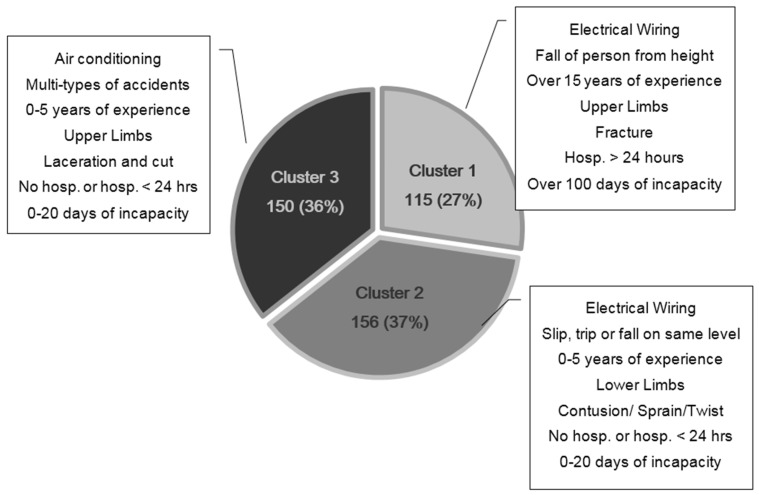
Cluster sizes of the three clusters of E&M accident cases.

Cluster analysis was conducted on the variables of E&M works-related accidents to identify groups with different pattern of accidents. Clustering is an effective method for segmenting the dataset in more homogeneous groups and for identifying the variables that may have a higher influence on injury type and accident severity. Clustering for accident case analysis is important in safety research to reveal characteristics of accident situations and injured workers for designing safety preventive measures in future. To assess complex relations between E&M accidents and accident outcome, seven key variables were selected for analysis (Table 1). Previous literature indicated that type of works, type of accident and worker’s length of experience are crucial features of construction accidents [37,41,42,43]. Thus, these variables were chosen for analysis. Moreover, variables related to the accident outcome such as body part injured, nature of injury, severity of injury and period of incapacity were also selected to form the cluster model. The 421 E&M accident cases were formed into three clusters (Figure 2). Schwarz’s Bayesian Criterion (BIC) or the Akaike Information Criterion (AIC) are the common clustering criteria to determine the optimum number of clusters. The Schwarz’s Bayesian Criterion (BIC) with silhouette was used to determine the final optimum number of clusters for the final cluster model as Vermunt and Magidson [44] pointed out that BIC is a more reliable criterion than (AIC) especially for large datasets. The final three-cluster model was described by the proportion of each variable in each cluster, which enables us to identify each cluster as a specific E&M accident situation. The profile of the three clusters is shown in Table 4.

#### 5.2.1. Cluster 1

Cluster 1 included 115 accident cases. This cluster consists of workers with more than 15 years of working experience in construction and involved in electrical wiring installation. A vast majority of accidents in this cluster were caused by ‘fall of person from height’ and resulted in upper limb fractures. Due to the higher severity of accident, the injured worker needed hospitalization for more than 24 h and suffered over 100 days of incapacity for work.

#### 5.2.2. Cluster 2

Cluster 2 included 156 accident cases, which represent 37% of the reported E&M accidents. Workers with less working experience (i.e., zero to five years) undertaking electrical wiring installation work are classified in cluster 2. ‘Slip, trip or fall on same level’ was the most common accident type in this cluster. Most of the victims suffered from contusion, sprain or twist of lower limbs. The injured workers suffered less severe injury with no hospitalization or with hospitalization for less than 24 h and not more than 20 days of incapacity for work.

#### 5.2.3. Cluster 3

Cluster 3 included 150 E&M accident cases with workers who were involved in air-conditioning installation works with zero to five years of working experience. The workers’ injuries were caused by multiple types of accidents such as exposure to fire, stepping on object. Common classifications for nature of injury were crushing, and laceration or cut on upper limbs. The victims of this cluster mostly did not require hospitalization or required hospitalization for less than 24 h and suffered incapacity for less than 20 days.

The cluster analysis results of the 421 E&M works-related accidents and their distribution in each cluster are summarized in Table 5. In terms of E&M trade, electrician accounted for the greatest number of accident cases. The current findings indicated that electrical wiring and air-conditioning installation works were the top two hazardous trades of E&M works, accounting for about 41% and 31% of all E&M accident cases respectively. From the analysis results, the variable of “type of accident” is the most important predictor of the formation of the clusters. E&M installation works result in various types of accidents. “Slip, trip or fall on same level” (*n* = 98) and “fall of person from height” (*n* = 87) were the most common types of E&M accident. Both accident types demonstrated a high frequency of upper and lower limbs injuries. Other types of accident (*n* = 99) which encompasses a range of miscellaneous accident types such as exposure to fire/burning, dust/foreign particle in eye, stepping on object, and crushing, etc. The patterns of injury nature and accident types varied substantially. For instance (Table 5), fracture was a major type of injury nature associate with ‘fall of person from height’. Contusion, sprain or twist were the main injury due to ‘slip, trip or fall on same level’. ‘Fall of person from height’ resulted in a higher number of fracture injuries indicating that falls from an elevation could generate more severe injuries and longer period of incapacity.

**Figure 3:**
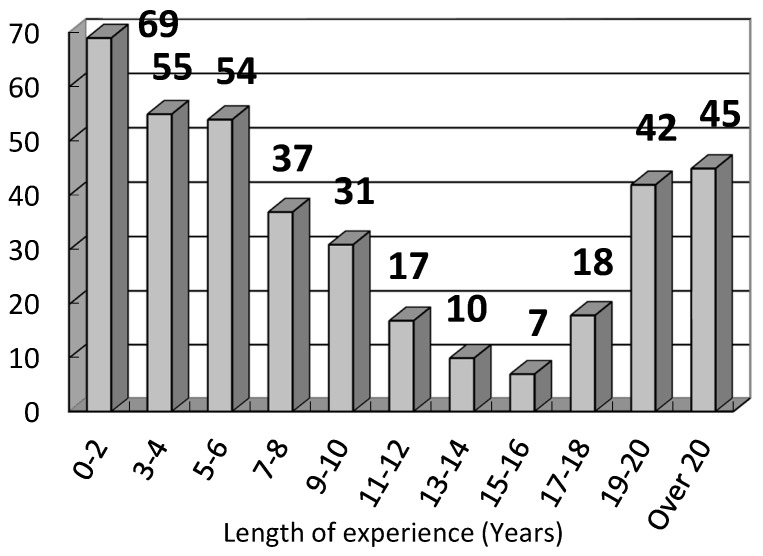
E&M accident data analysed by length of experience of the injured worker.

The cluster model revealed the relationship of accident types and a comprehensive set of factors associated with the accident. The first cluster identified was electrical wiring workers with more than 15 years of working experience in construction. This is not surprising that electricians are the most accident-prone E&M workers as they need to work at height and involve electrical hazards. However, it is unexpected that workers with more experience were more prone to accidents in this cluster. It undermines the concept that safety attitude is normally built up through experience. The E&M accident data was further analyzed by the length of experience of the injured worker and illustrated in Figure 3. It is obvious that a downward trend of the number of accidents was revealed with the increase of length of the worker’s experience. It is rather surprising that the number of accidents of workers with over 19 years of experience was significantly higher than expected. A U-shape relationship was discovered between year of experience and number of E&M accidents, indicating that year of experience can increase the safety awareness of workers for a certain period of time, but became less of an advantage over a certain years of experience. The safety awareness of experienced E&M workers may reduce if they take safety lightly with the increase of experience. The interviewees of Choudhry and Fang [18] revealed that workers with more site experience did not feel comfortable following proper safety procedures and were not afraid of getting hurt.

The vast majority of accidents in this cluster were caused by ‘fall of person from height’ and result in upper limb fractures. These accidents mainly involve falls from ladders or working platforms. Serious accidents may happen when the worker falls due to the sudden collapse of the ladder or working platform or loses his balance while conducting installation works. Numerous studies supported that fall of person from height causes a greater number of severe and even fatal accidents in the construction sector [37,45,46]. The injured worker needs hospitalization more than 24 h and suffers over 100 days of incapacity for work. Hence, the long period of incapacity of workers incurred extra financial costs, including loss due to absence from work of the injured worker, inefficiency after resuming work of the injured worker, medical expenses, fines and legal expenses and loss due to damaged material or finished work, etc. [47,48].

Similar to cluster 1, the second cluster had the largest group of accident cases consisting of electrical wiring installation workers. However, the injured workers in this cluster had relatively less working experience (i.e., zero to five years). The research of Choudhry and Fang [18] revealed that young workers with less experience are more prone to accidents. The research further pointed out that the most effective safety training for new construction workers is learned by doing or by gaining experience. New workers become more aware of construction safety with the accumulation of working experience. The workers with more experience have more job knowledge, skills and patience [41]. Most of the accidents in this cluster were caused by ‘slip, trip or fall on same level’ and resulted in contusion, sprain or twist of lower limbs. The injured workers in this cluster suffered less severe injury with no hospitalization or hospitalization for less than 24 h and not more than 20 days of incapacity for work. The findings are in line with the research of Lipscomb et al. [49] that individuals performing electrical wiring works suffered slip/trip injury at a significant higher rate than other types of work. Major slip/trip injuries were related to soft tissue injuries such as sprains, strains or contusions and more commonly led to injuries of lower extremity [49,50]. Slips and trips are regarded as one of the most significant type of construction accidents [49,51,52]. Lipscomb et al. [49] found that environmental factors, such as slippery or uneven working surfaces, weather and lighting, were the most frequent and common contributor to slip/trip injuries. Besides, poor housekeeping and human factors (i.e., lapse of attention, carelessness or rush to finish work, etc.) also contribute to these accidents [49,53].

The third cluster covered workers who were involved in air-conditioning installation works with zero to five years of working experience. Camino López et al. [37] indicated that construction workers with a short period of services suffered a higher percentage of accidents. This cluster revealed that workers of air-conditioning works are prone to E&M accidents. The research findings also indicated that air-conditioning installation and maintenance works were the second most risky form of E&M works. Most of the accidents occurred in Air Handling Unit (AHU) room or AC plant room for carrying out installation or maintenance of the AC system. A combination of project complexity, poor working conditions and hazardous nature of work is leading to variety types of accident. This cluster group included multiple types of accidents such as exposure to fire, hand tool accident, stepping on object and crushing, etc. These accident types are easily overlooked but the research findings show that multiple types of accidents contribute a significant number of accident cases. The accidents in this cluster are mainly less severe and caused laceration and cut on upper limbs. The victims of this cluster mostly did not require hospitalization or required hospitalization for less than 24 h and led to incapacity for less than 20 days.

Other factors for E&M accidents were also evaluated (Table 6). Improper procedure and poor housekeeping were the top two unsafe conditions, whereas lapse of attention was the key unsafe action among the E&M accident cases. Carelessness or not concentrating were the most significant personal factor of accidents. According to Zhou et al. [54], major types of improper construction procedures refer to failure to operate in accordance with safety specifications and construction guidelines. For example, the electrical workers fail to de-energize or lock out electrical circuits for electrical wiring works. Ignorance of safety procedures and proper construction process may substantially increase the probability of E&M accidents. Besides, Bentley [55] and Lipscomb et al. [49] advocated that housekeeping or orderliness highly influence workers’ exposure to ‘slip, trip or fall’ hazards. E&M works undertaken on slippery or uneven floor may cause ‘slip, trip or fall’ accidents if workers are not fully concentrated on their work.

## 6. Recommendations

Three clusters encapsulating a total of 421 E&M accident cases have been analyzed and discussed in this paper. A series of recommendations are suggested for better allocation of safety resources to enhance the safety performance of E&M works regardless of RMAA or new construction works.

### 6.1. Enhance Training and Supervision to High Risk Group

It would be most effective to formulate targeted safety measures for the high-risk groups (i.e., workers for electrical installation works, young E&M workers and workers with zero to five years of experience and more than 19 years of experience). The findings of the current study indicate that 55% of E&M accidents occurred in this two experience groups. The E&M workers with less experience may not be competent to identify the risks involved in E&M works. The safety awareness of experienced workers may decrease and they may overlook the associated hazards. It is recommended that the high-risk workers should receive more training. Suitable training should be provided to workers before they start working, and before being assigned to a job which requires new skill and after any deficiency is detected [56]. Lee [57] promoted the introduction of safety orientation programmes for new workers to impress their safety awareness.

With adequate safety training, the competent safety person would be responsible for identifying safety hazards, checking safety equipment, and reminding the corresponding workers constantly. Establishment of appropriate safety program and safety supervision by competent safety personnel is essential in protecting workers from workplace hazards [58,59,60,61]. The safety personnel should closely supervise the workers involved in high-risk activities such as electrical wiring works and work at height, etc., ensuring proper use of personal protective equipment and correcting any unsafe action and condition.

### 6.2. Safety Measures for Working at Height

A vast majority of accidents in cluster one are caused by ‘fall of person from height’ and result in severe fracture injuries. After analyzing the features of cluster one, it is suggested that safety measures should be conducted to prevent reoccurrence of fall accidents. E&M works often involve the use of ladders. Ladder was found to be the most common agent involved in fall accidents. Workers were injured with ladders being used to perform installation tasks or gain access to areas in most accident cases. Ladder is designed only for temporary use or provide access to different elevations. Workers are prevented from performing prolonged tasks on ladders [62]. Wong et al. [63] revealed that poor maintenance condition of ladders and improper use of ladders were the top causes of fall accidents when using ladders. It also indicated that “better control” and “enhance monitoring” of using ladders are the key measures to ensure the safe use of ladder, thus, minimize fall accidents from ladders. Safety checking system for equipment (i.e., ladders, hand tools, safety harness, etc.) should be established to ensure all the equipment are in safe working order. Safe means of support such as platform ladder and working platform should be provided for access or work at height.

### 6.3. Improve Housekeeping

For the second cluster, housekeeping and the conditions of the construction site should be improved to prevent the accidents caused by ‘slip, trip or fall on same level’. Numerous studies indicated that tidy and safe working environment is one of the most important factors associated with good performance of construction safety [55,58,64]. In the continuously changing work environment of E&M works, frequent and regular housekeeping and walk-through assessments by safety personnel would be important to identify hazards such as slippery or uneven working surface [51]. These strategies could make substantial contributions in preventing accident of ‘slip, trip or fall on same level’.

### 6.4. Proper Working and Safety Procedures

Improper procedure was regarded as one of the major unsafe conditions among the accident cases. Apparently, improvement of E&M works’ safety can be achieved by ensuring proper working and safety procedures. Negligence of safety procedures and proper construction processes may substantially increase the probability of E&M accidents. Improper working procedures such as failing to de-energized before electrical works or failing to conduct lock-out and tag-out procedures leads to a risk of electrical shock. Safety personnel or site foreman are required to ensure the provision and use of correct equipment and implementation of appropriate construction procedures and methods [54,65].

Wilson and Koehn [14] pointed out that safety management is one of the major methods to control safety policies, and working procedures relating to a construction site. Proper working and safety procedures for E&M tasks can be delivered through safety training. Safety education transfers the concepts of the importance of work safely and how an unsafe act will seriously affect them.

### 6.5. Implement Risk Assessment Process

E&M installation works are regarded as high-risk operations in construction process. Due to the tight working schedule of E&M works in new construction works, the process of risk assessment may be neglected. Lack of risk assessment at the workplace may lead to E&M accidents. For new construction works, it is important to conduct risk assessment by safety personnel at an early stage. This helps to identify hazardous works such as confined working spaces, work at height and corresponding safety equipment, and to estimate the cost for safety investment (e.g., provision of working platform and scissor lift). It is also required to make a risk assessment of health and safety to employees and others who are exposed on construction sites, especially for specific hazards (e.g., work at height, hazardous substance, manual handing, and use of plant, etc.). Risk assessment is often ignored in RMAA works due to the short construction period and limited safety resources. An appropriate risk assessment for E&M RMAA may identify people who are being affected by the activity, the requirements of personal protective equipment, suggested additional risk control measures, and any applicable guidance related to the operation. Moreover, it is highly recommended to conduct permit-to-work systems in situations where the nature of work is complicated, the scope of work is wide, or there are energised parts in the switchgear/switchboard when the work is carried out. The permit-to-work system should include the following: (1) risk assessment of the task; (2) identifying the hazards; (3) define safety precautions; (4) strictly implementing the system; and (5) close monitoring of the system.

## 7. Conclusions

The significance of this paper lies in investigating the link between E&M works and related accidents in public sector projects based on a rich set of factors such as working environment, demographics, unsafe factors, and personal factors, etc. The clustering of accident cases provides an understanding of the relationship between type of E&M works, type of accident, severity and body part of injury, experience of workers, and other unsafe factors. The accident analysis is vital for industry practitioners and relevant Government Departments to enhance the safety performance of public sector projects. This paper analysed 421 accident cases from the Electrical and Mechanical Services Department (EMSD) and the Architectural Services Department (ArchSD) of the Hong Kong Government in a 15 years period. A two-step cluster analysis was applied to identify cluster groups in a heterogeneous E&M accident data set. Three clusters of E&M works-related accidents were identified. Cluster one was electricians with over 15 years of experience and prone to ‘fall of person from height’, the accidents caused upper limbs fracture with over 100 days of incapacity for work. Cluster two was electricians with zero to five years of experience and prone to ‘slip, trip or fall on same level’ causing lower limbs contusion, sprain or twist with 0–20 days of incapacity. Cluster three was air-conditioning workers who acquired 6–10 years of experience and were prone to multiple types of accidents causing laceration and cut on upper limbs and 0–20 days of incapacity. The results of the cluster analysis provide interesting insights into various E&M works-related accidents. Recommendations to avoid recurrence of similar occurrence have also been provided. Although the analysis of E&M work-related accidents were from Hong Kong, the research findings are believed to be applicable to other countries as well. The research outcomes would be essential for safety managers to estimate the associate risks of accident occurrence and injury characteristics and pay more attention or allocate more safety resources to prevent E&M-works related accidents and to achieve a safer working environment.

## Figures and Tables

**Table 1:** Variables in cluster analysis.

Variable	Values
Gender	Male; Female
Age	24 or below; 25–34; 35–44; 45–54; over 54
Day of week	Beginning of the week (Monday–Tuesday), middle of the week (Wednesday–Thursday), end of the week (Friday), weekends (Saturday–Sunday)
Season	Spring (March–May); Summer (June–August); Autumn (September–November); Winter (December–February)
Type of accident *	Injured whilst lifting or carrying; Slip, trip or fall on same level; Fall of person from height; Contact with electricity; Struck by moving or falling object; Striking against object; Other (e.g., contact with moving machinery, exposure to fire, stepping on object, etc.)
Trade of E&M works *	Air-conditioning; Electrical wiring; Lift and escalator; Fire services; Plumbing and Drainage
Type of works	New construction works; Repair; Maintenance; Minor Alteration and Addition (RMAA) works
Imported labourer	Yes; No
Length of experience * (no. of years)	0–5; 6–10; 11–15; over 15; unknown
No. of month at this site (no. of months)	0–3; 4–6; 7–9; 10–12; over 12; unknown
Body part injured *	Head (e.g., Skull/scalp, eye, ear, face, etc.); Neck and Trunk (e.g., Back, chest, waist, etc.); Upper limbs (Finger, hand/palm, shoulder, elbow, etc.); Lower limbs (e.g., Hip, ankle, foot/toe, etc.)
Injury nature *	Laceration and cut; Crushing; Fracture; Contusion/sprain/twist; Electric shock; Others (e.g., multiple injuries, burn, abrasion, freezing, etc.)
Severity of injury *	No hospitalization or hospitalization <24 h, Hospitalization >24 h, Fatal, Pending confirmation
Period of Incapacity * (no. of days)	0–20; 21–40; 41–60; 61–80; 81–100; over 100
Agent involved	Portable power or hand tools; Material being handled or stored; Ladder or working at height; Floor, ground, stairs or any working surface; Electricity supply, wiring apparatus or equipment; Others (e.g., gas, pipe, machinery, scaffolding, etc.)
Unsafe action	Use unsafe equipment/use equipment unsafely; Adopting unsafe position or posture; Failure to use personal protective equipment; Use of unsuitable access; Lapse of attention; Others (e.g., operating with authority, failure to secure objects, etc.)
Unsafe condition	Improper procedure; Poor housekeeping; Inadequate working space/platform; Inadequate tool and protective equipment; Others (e.g., lack of warning system, improper illumination, improper ventilation, etc.)
Personal factor	Incorrect attitude/motive; Lack of knowledge or skill; Fatigue/exhaustion; Carelessness/Not Concentrating; Others (e.g., physical defects, unsafe act by another person, etc.)

* Key variables used in the cluster analysis.

**Table 2:** Background information of the electrical and mechanical (E&M) works-related accident cases.

Information	Category	Frequency	Percentage
Data source	Electrical and Mechanical Services Department (EMSD)	170	40.4%
	Architectural Services Department (ArchSD)	251	59.6%
Gender	Male	418	99.3%
	Female	3	0.7%
Age	24 or below	60	14.3%
	25–34	127	30.2%
	35–44	97	23.0%
	45–54	101	23.9%
	Over 54	36	8.6%
Imported Labourer	Non-imported labourer	411	97.6%
	Imported labourer	6	1.4%
	Unknown	4	1.0%
Type of works	New construction works	113	26.8%
	Repair, maintenance, minor alteration, and addition (RMAA) works	308	73.2%
Season	Spring	116	27.6%
	Summer	134	31.8%
	Autumn	85	20.2%
	Winter	86	20.4%
Day of week	Beginning oft he week	152	36.1%
	Middle of the week	120	28.5%
	End of the week	71	16.9%
	Weekends	78	18.5%

**Table 3:** Experience of the injured worker.

Information	Category	Frequency	Percentage
Length of experience (no. of years)	0–5	146	34.7%
	6–10	100	23.8%
	11–15	42	9.9%
	Over 15	97	23.0%
	Unknown	36	8.6%
No. of months at this site (no. of months)	0–3	220	52.4%
	4–6	62	14.7%
	7–9	40	9.5%
	10–12	24	5.7%
	Over 12	36	8.5%
	Unknown	39	9.2%

**Table 4:** Summary of key variables used in the cluster analysis and their distribution in each cluster.

Variable	Category	Cluster 1 (*n* = 115)	Cluster 2 (*n* = 156)	Cluster 3 (*n* = 150)	Total (*N* = 421)	*p*-Value
Trade of E&M works	Air-conditioning	24	43	65	132	<0.001
	Electrical wiring	73	65	33	171	
	Lift and escalator	0	16	30	46	
	Fire services	11	19	9	39	
	Plumbing and drainage	7	13	13	33	
Type of accident	Injured whilst lifting or carrying	4	26	23	53	<0.001
	Slip, trip or fall on same level	21	50	27	98	
	Fall of person from height	65	21	1	87	
	Contact with electricity	0	0	9	9	
	Struck by moving or falling object	8	18	11	37	
	Striking against object	2	11	25	38	
	Others (e.g., exposure to fire, stepping on object etc.)	15	30	54	99	
Length of experience (no. of years)	0–5	32	65	49	146	<0.05
	6–10	27	39	34	100	
	11–15	7	16	19	42	
	Over 15	40	25	32	97	
	Unknown	9	11	16	36	
Body part injured	Head	16	48	5	69	<0.001
	Neck and Trunk	13	47	0	60	
	Upper limbs	45	5	140	190	
	Lower limbs	41	56	5	102	
Injury nature	Laceration and cut	15	20	64	99	<0.001
	Crushing	3	4	7	14	
	Fracture	73	12	27	112	
	Contusion/Sprain/Twist	15	96	27	138	
	Electric shock	0	0	9	9	
	Others (e.g., multiple injuries, burn, abrasion, freezing etc.)	9	24	16	49	
Severity of injury	No hospitalization or hosp. < 24 h	16	128	127	271	<0.001
	Hosp. > 24 h	99	20	21	140	
	Fatal	0	4	0	4	
	Pending confirmation	0	4	2	6	
Period of Incapacity (no. of days)	0–20	34	121	112	267	<0.001
	21–40	18	15	16	49	
	41–60	3	6	3	12	
	61–80	4	4	1	9	
	81–100	3	1	2	6	
	Over 100	53	9	16	78	

Notes: The null hypothesis of significant difference is rejected if the *p*-value from the Chi-square test is less than the significance level at five percent. It indicates that there are statistically significant differences among the variables across the three clusters.

**Table 5:** Frequency distribution of injury nature and severity with respect to the major types of accident.

	Slip, Trip or Fall on Same Level	Fall of Person from Height
**Major nature of injury**		
Laceration and cut	14	5
Fracture	26	53
Contusion/Sprain/Twist	43	23
**Severity of injury**		
No hospitalization or hosp. < 24 h	72	30
Hosp. > 24 h	25	56

**Table 6:** Summary of variables related to unsafe personal or environmental factors and their distribution in each cluster.

Variable	Category	Cluster 1 (*n* = 115)	Cluster 2 (*n* = 156)	Cluster 3 (*n* = 150)	Total (*N* = 421)
Agent involved	Portable power or hand tools	5	10	20	35
	Material being handled or stored	13	28	29	70
	Ladder or working at height	55	25	4	84
	Floor, ground, stairs or any working surface	13	54	42	109
	Electricity supply, wiring apparatus or equipment	3	3	13	19
	Others (e.g., gas, pipe, machinery, scaffolding, etc.)	26	36	42	104
Unsafe action	Use unsafe equipment/use equipment unsafely	6	10	13	29
	Adopting unsafe position or posture	22	30	20	72
	Failure to use personal protective equipment	10	22	26	58
	Use unsuitable access	11	10	8	29
	Lapse of attention	40	52	50	142
	Others (e.g., operating with authority, failure to secure objects, etc.)	26	32	33	91
Unsafe condition	Improper procedure	40	45	39	124
	Poor housekeeping	31	60	25	116
	Inadequate working space/platform	7	11	13	31
	Inadequate tool and protective equipment	12	11	23	46
	Others	25	29	50	104
Personal factor	Incorrect attitude/motive	12	15	19	46
	Lack of knowledge or skill	11	10	9	30
	Fatigue/exhaustion	3	2	5	10
	Carelessness/Not concentrating	78	98	90	266
	Others (e.g., physical defects, unsafe act by another person, etc.)	11	31	27	69

## Abbreviations

ArchSD	Architectural Services Department
DEVB	Development Bureau
E&M	Electrical and Mechanical
EMSD	Electrical and Mechanical Services Department
PCSES	Public Works Programme Construction Site Safety and Environmental Statistics
RMAA	Repair, Maintenance, Minor Alteration, and Addition
